# Where to Biopsy to Detect *Helicobacter pylori* and How Many Biopsies Are Needed to Detect Antibiotic Resistance in a Human Stomach

**DOI:** 10.3390/jcm9092812

**Published:** 2020-08-31

**Authors:** Maxime Pichon, Cong Tri Tran, Gaëtan Motillon, Charlotte Debiais, Sylvain Gautier, Marie Aballea, Julie Cremniter, Philippe Vasseur, David Tougeron, Magali Garcia, Martine Garnier, Charles Bodet, Jean Pierre Faure, Christophe Burucoa

**Affiliations:** 1 CHU de Poitiers, Département des Agents Infectieux, Laboratoire de Bactériologie, 86021 Poitiers, France; ct.tran@ch-aurillac.fr (C.T.T.); gaetan.motillon@gmail.com (G.M.); charlottedebiais@gmail.com (C.D.); sylvain.gautier@uvsq.fr (S.G.); marie.aballea@chu-poitiers.fr (M.A.); julie.cremniter@chu-poitiers.fr (J.C.); magali.garcia@chu-poitiers.fr (M.G.); 2 Université de Poitiers, EA4331, LITEC, 86022 Poitiers, France; philippe.vasseur@chu-poitiers.fr (P.V.); David.tougeron@chu-poitiers.fr (D.T.); martine_garnier@hotmail.com (M.G.); charles.bodet@univ-poitiers.fr (C.B.); 3 Université de Poitiers, INSERM U1070, 86022 Poitiers, France; 4 CHU de Poitiers, Service d’hépato-gastro-entérologie, 86021 Poitiers, France; 5 CHU de Poitiers, Service de Chirurgie Viscérale Digestive, 86021 Poitiers, France; Jean-pierre.faure@chu-poitiers.fr

**Keywords:** *Helicobacter pylori*, antimicrobial resistance, genetic diversity, gastric colonization, sleeve gastrectomy

## Abstract

This study aims to determine the gastric distribution, density, and diversity of *Helicobacter pylori* infection. Subtotal resection of the stomachs of three *H. pylori*-infected and asymptomatic obese patients were collected after a sleeve gastrectomy. Distribution and density of *H. pylori* were determined using culture and RT-PCR on multiple gastric sites (88, 176, and 101 biopsies per patient). Diversity of *H. pylori* strains was studied using antibiotic susceptibility testing, random amplified polymorphism DNA (RAPD) typing and *cagA* gene detection on single-colony isolates (44, 96, and 49 isolates per patient). *H. pylori* was detected in nearly all analyzed sites (354/365 biopsies, 97%). Antral density was higher in one patient only. The three stomachs were almost exclusively infected by an antibiotic-susceptible strain. One clarithromycin-resistant isolate in one biopsy was detected in two stomachs (1/44 and 1/49 isolates), while in the third one, eight (8/96 isolates) metronidazole-resistant isolates were detected. DNA typing showed infection with *cagA*-negative strains for one patient, *cagA*-positive strains for a second patient and the third patient was infected with two different strains of distinct *cagA* genotypes. Infection with *H. pylori* is shown to spread to the whole surface of the stomach, but a possibility of minor sub-population of antibiotic-resistant clones, undetectable in routine practice.

## 1. Introduction

*Helicobacter pylori* (*H. pylori*) is a Gram-negative bacterium, chronically infecting the gastric mucosa of about half the human population worldwide [1,2]. Infection is mostly acquired during childhood and persists for life [3]. *H. pylori* lives either as a planktonic population within the protective mucus layer or are attached to the epithelial surface and persist as microcolonies on the epithelial surface of the gastric glands [4]. This infection, mainly asymptomatic, is responsible for chronic gastritis which may progress to peptic ulcer, adenocarcinoma, and mucosa-associated lymphoid tissue (MALT) lymphoma [1,2,5]. The *cag* pathogenicity island is responsible for the injection of the CagA bacterial protein into the gastric epithelial cell, enhancing inflammation and carcinogenesis. Indications for diagnosis and treatment of *H. pylori* infections have been considerably extended since its discovery in 1982 [6]. Due to frequent antibiotic use, in general, and in particular for the eradication of *H. pylori*, the number of resistant strains has increased rapidly worldwide [7,8,9,10]. The emergence of resistance is responsible for an increase of therapeutic failures, justifying the need for gastric biopsies to guide the treatment. European guidelines highlight the importance, before the first treatment, of culture and antimicrobial susceptibility testing (AST) in populations known to carry high resistance to clarithromycin [11]. Strategy based on AST or PCR searching for resistance-associated mutations is, therefore, more effective than primarily empirical treatment strategy [12]. Nevertheless, no recommendation on the number and location of gastric biopsies required is available. While earlier studies have shown the antral predominance of bacterial colonization, justifying the recommendation of antral biopsies for bacteriological diagnosis, these studies have only analyzed a small number of biopsies (2 to 12) per patient and have often been based on histological studies (and not on culture or PCR) [13,14,15,16,17,18]. As the distribution of bacteria in the stomach could be heterogeneous, the need for several biopsies is justified, but during routine practice, only one antral and one corpus biopsies are analyzed by the bacteriological laboratory.

Numerous studies have demonstrated that the gastric mucosa can be infected by both susceptible and resistant subpopulations of the same bacterial strain (defined as “hetero-resistance” or “mixed infection”) or by different strains with distinct genetic profiles (defined as “multiple infection”) [19,20,21,22]. These specific situations are difficult to detect in routine practice (especially when the number of biopsies is limited), exposing to a risk of treatment failure [20].

Laparoscopic sleeve gastrectomy is bariatric surgery which consists of a subtotal resection (−3/4) of the stomach, allowing to gain further insight into gastric infection by *H. pylori* [23]. These resected tissues have already been used to isolate gastric stem cells or gastric cells to develop in vitro models of *H. pylori* infection [24,25,26].

The aim of this study was to describe the gastric distribution, density, and diversity of *H. pylori* in the stomach collected after sleeve gastrectomy to determine number and location of biopsies needed to detect presence and resistance of *H. pylori*.

## 2. Methods

### 2.1. Patients

During a two-year period (2012–2014), seven obese patients (body mass index > 40 kg/m) were detected infected by *H. pylori* (endoscopy diagnosis performed two months before the intervention). Five accepted to participate in the study, but one of them took antibiotics before surgery, and one stomach was contaminated by a fungus, then, three patients infected by *H. pylori* were included. They underwent sleeve gastrectomy (laparoscopic resection of the three-quarters of the stomach associated with concomitant stapling all along the greater curvature of the stomach) [22]. Doing so, the anatomical pieces represent nearly a complete stomach (except for the lesser curvature).

### 2.2. Collection of Gastric Biopsies 

The anatomical pieces of the three patients were collected during surgery and immediately transported to the laboratory in saline buffer at room temperature. Each specimen was sliced into two equal parts (the lower part was designed as “antrum”, the upper part as “corpus”) and spread on a glass plate over a sterile corkboard, mucosa side up, then covered with a sterile plastic plate perforated with 2 mm-spaced holes of 6-mm in diameter (*n* = 195). Biopsies were performed through the plastic holes with 4-mm sterile punches (Stiefel, Wachtersbach, Germany). Between each biopsy, punches were washed in 90% alcohol then rinsed in sterile saline buffer, ensuring the absence of cross-contamination between samples. Collection of biopsies was processed within four hours. After sampling, biopsies were immediately frozen at −80 °C in 100 µL Brucella liquid broth supplemented with 20% glycerol (bioMérieux, Marcy-l’étoile, France).

### 2.3. Analysis of Gastric Biopsies 

Gastric distribution and density of *H. pylori* in randomly selected biopsies to cover the different regions of the anatomical piece (*n* = 88 per stomach) were analyzed by culture and quantitative real-time PCR (qPCR). In the absence of a previous comparable study, the number of biopsies studied was decided empirically as a compromise between topographic representativeness and workload. Density was expressed in colony-forming-units (CFU) per biopsy for culture and in equivalent eCFU per biopsy for qPCR.

#### 2.3.1. Culture and AST

Biopsies were thawed, then processed as regular biopsies for routine *H. pylori* culture detection. Biopsies were shred with a scalpel in a sterile Petri dish. One half of the homogenized grinding material was spread on Columbia 10% horse’s blood agar supplemented with antibiotics (Skirrow’s supplement: Trimethoprim 5 mg/L, Amphotericin B 2 mg/L, Vancomycin 10 mg/L, and Polymixin 50 mg/L) (Oxoid, Basingstoke, UK), and then incubated at 35 °C in a humid microaerobic atmosphere in an airtight jar (CampyGen*, Oxoid, ThermoFisher Scientific, Waltham, MA, USA). Agar plates were examined at day 3 (D3), D6, D9, D12 and D15. *H. pylori* isolates were identified as positive for urease activity and spiral shape morphology on Gram stain. When positive, CFUs were numbered.

AST for amoxicillin, clarithromycin, tetracycline, metronidazole, and levofloxacin was performed using an E-test method (bioMérieux, Marcy-l’étoile, France) on Columbia agar plates supplemented with 10% horse blood (Oxoid, ThermoFisher Scientific, Waltham, MA, USA) as recommended by the CA-SFM EUCAST [27]. Briefly, a 3-McF inoculum was spread on agar plates. The minimal inhibitory concentration (MIC) cut-offs to consider resistance were 0.12 mg/L for amoxicillin, 0.50 mg/L for clarithromycin, 1 mg/L for tetracycline and levofloxacin, and 8 mg/L for metronidazole.

#### 2.3.2. DNA Extraction and Molecular Biology Analyses

The other half of the grinding material from biopsies was suspended in 350 µL of lysis buffer MagNa Pure Bacteria (MagNa Pure Compact Nucleic Acid Isolation Kit I, Roche, Meylan, France) with 50 µL of proteinase K 20 mg/L (EU0090, Euromedex, Souffelweyersheim, France), and incubated at 65 °C for 3 h. Extraction was processed in a Roche Diagnostic MagNa Pure Compact system (Roche). Elution was performed in 100 µL elution buffer, and extracts were then frozen at −80 °C until analysis.

To extrapolate quantitative results for biopsies, a standard curve for PCR was implemented using ten-fold geometric serial dilutions (1 × 10^−1^ to 1 × 10^−7^) of a CFU-numbered suspension of a 48 h-cultured *H. pylori* J99-type strain [28,29]. Presence of *H. pylori* was determined by detecting the *glmM* gene specific for this bacterial species. Amplification of this gene was performed using the following primers: UreC5 (forward) 5′-AGC-GCT-TCC-CTC-ACT-GGC-ATA-3′ and UreC6 (reverse) 5′-TCT-GTT-TCG-AAA-AAA-GCG-AT-3′. Detection was performed using the labeled probe (FAM/TAMRA): S-ureC (5′) 6-FAMTGA-TBI-AAA-TAG-GGC-CTA-TGC-CTA-CCC-C (3′) 3-TAMRA. PCR reactions were performed in a final volume of 25 µL (12.5 µL of Premix Ex Taq (TaKaRa, Shiga, Japan), 5 µL of DNA extract for biopsies or 1 µL for strains in culture, 0.15 µL of each primer at 12.5 µM and 0.3 µL of the probe at 5 µM. Amplification was performed on the SmartCycler (Cepheid, Sunnyvale, CA, USA) according to the following protocol: Initial denaturation at 95 °C for 20 s followed by 45 cycles (denaturation for 5 s at 95 °C, annealing for 10 s at 55 °C and elongation at 72 °C for 20 s, including the measure of fluorescence). Data were analyzed by the manufacturer’s software (Cepheid). The amount of eCFU was numbered using the calibration curve. Absence of PCR inhibitors was assessed by detection of a 110-bp-long fragment of a human housekeeping gene (β-globin) [28].

Detection and identification of mutations associated with clarithromycin resistance (A2142G, A2142C and A2143G) were performed using a multiplex real-time Scorpion PCR assay [29]. Each run was validated using positive (DNA extracts of sequenced strains) and negative (molecular-quality distilled water) controls. As Scorpion PCR is easier, faster and cheaper than E-test, more isolates were analyzed with Scorpion PCR [29].

Random amplified polymorphism DNA reaction (RAPD-PCR) was carried out as previously described on a Perkin-Elmer GeneAmp PCR system 2400 thermal cycler (Perkin-Elmer Cetus, USA). Two different primers were used: Primer 1254 (5′-CCG-CAG-CCA-A-3′) and primer 1247 (5′-AAG-AGC-CCG-T-3′) [30]. Reaction mixes (total volume of 100 µL) contained 1 µL of DNA extract, 3 mM MgCl_2_, primer 1254 and primer 1247 at a concentration of 0.2 µM, 2.5 U of Eurotaq DNA polymerase (Eurogentec, Liège, Belgium), dinucleotide triphosphate (Eurogentec) at a concentration of 0.2 µM, 10 mM Tris-HCl (pH 8.3), and 50 mM KCl. The cycling program was an initial cycle (94 °C for 2 min, 37 °C for 1 min, and 72 °C for 4 min) then followed by 29 amplification cycles (94 °C for 2 min, 37 °C for 3 min, and 72 °C for 7 min) [30]. After PCR, amplification products (20 µL) were separated on 2% agarose gels. Criteria defined by Tenover et al. were used for interpreting RAPD banding patterns, considering indistinguishable and closely related RAPD banding patterns and bacterial infection with more than one RAPD banding pattern in the isolates of one patient as a single-strain or multiple-strain infection, respectively [31]. 

Finally, determination of *CagA* status was performed using a real-time PCR [31].Briefly, primers were F1 (forward) 5′-GAT-AAC-AGC-CAA-GCT-TTT-GAG-G-3′ and B1 (reverse) 5′-CTG-CAA-AAG-ATT-GTT-TCG-CAG-A-3′. Thermocycler conditions were 5 min at 95 °C followed by 45 amplification cycles (denaturation for 20 s at 95 °C, annealing for 15 s at 57 °C and elongation at 72 °C for 20 s). 

### 2.4. Statistical Analysis

Categorical data were analyzed by the Chi-square or Fisher’s exact test, while continuous variables were analyzed by the Student t-test. Spearman’s rank coefficient correlation was calculated to test the strength of association between two ranked variables. *p* values < 0.05 were considered as statistically significant.

### 2.5. Ethics

Designed with respect to the corresponding sections of the “World Medical Association Declaration of Helsinki-Ethical Principles for Medical Research Involving Human Subjects”, this study was approved by the local ethical board (“Comité de Protection des Personnes” (CPP) Ouest III, protocol #09.10.23) and written informed consent was obtained. 

## 3. Results

### 3.1. Characteristics of Included Patients

The patients were female, aged 29–42 years, with a BMI ranging from 43 to 49 kg/m^2^, strictly no digestive symptoms, and without macroscopic lesion during pre-surgery endoscopic examination (Table 1). Histopathological examination of five gastric biopsies showed chronic active gastritis with neutrophilic infiltration without precancerous lesion (no metaplasia nor dysplasia). *H. pylori* was isolated by culture from gastric biopsies. For each patient, one or two pooled biopsies were analyzed by culture and PCR. Two patients were exempt of any antimicrobial resistance, and the third showed resistance to clarithromycin (MIC 6 mg/L, mutation A2143G found using Scorpion PCR) and to metronidazole (MIC > 256 mg/L) in the unique biopsy collected in the lesser curvature. All baseline characteristics were summarized in Table 1.

### 3.2. Gastric Distribution and Density of H. pylori 

For patient A, 88 biopsies (antrum, *n* = 42 and corpus, *n* = 46) were cultured. Density of infection ranged from 0 CFU/biopsy (*n* = 8 biopsies, 4 antrum and 4 corpus) to 246 CFU/biopsy (mean 30 CFU/biopsy) (Table 2). For patient B, 176 biopsies (antrum, *n* = 90 and corpus, *n* = 86) were tested by culture. Density of infection ranged from 0 CFU/biopsy (*n* = 1, corpus biopsy) to 2800 CFU/biopsy (mean 333 CFU/biopsy). For patient C, 101 biopsies (antrum, *n* = 52 and corpus, *n* = 49) were tested by culture. Density of infection ranged from 0 CFU/biopsy (2 antrum biopsies) to 2800 CFU/biopsy (mean 373 CFU/biopsy).

For patient A, qPCR was performed on 90 biopsies (antrum, *n* = 44 and corpus, *n* = 46). Density of infection ranged from 0 eCFU/biopsy (*n* = 1, antrum biopsy) to 2.6 × 10^4^ eCFU/biopsy (mean 2.1 × 10^4^ eCFU/biopsy). For patient B, qPCR was performed on 177 biopsies (antrum, *n* = 90 and corpus, *n* = 87). Density of infection ranged from 0 eCFU/biopsy (3 antrum and 1 corpus biopsies) to 2.3 × 10^6^ eCFU/biopsy (mean 8.8 × 10^4^ eCFU/biopsy). For patient C, qPCR was performed on 103 biopsies (antrum, *n* = 53, and corpus, *n* = 50). Density of infection estimated ranged from 0 eCFU/biopsy (6 antrum and 8 corpus biopsies) to 2.3 × 10^4^ eCFU/biopsy (mean 1.1 × 10^3^ eCFU/biopsy).

**Figure 1:**
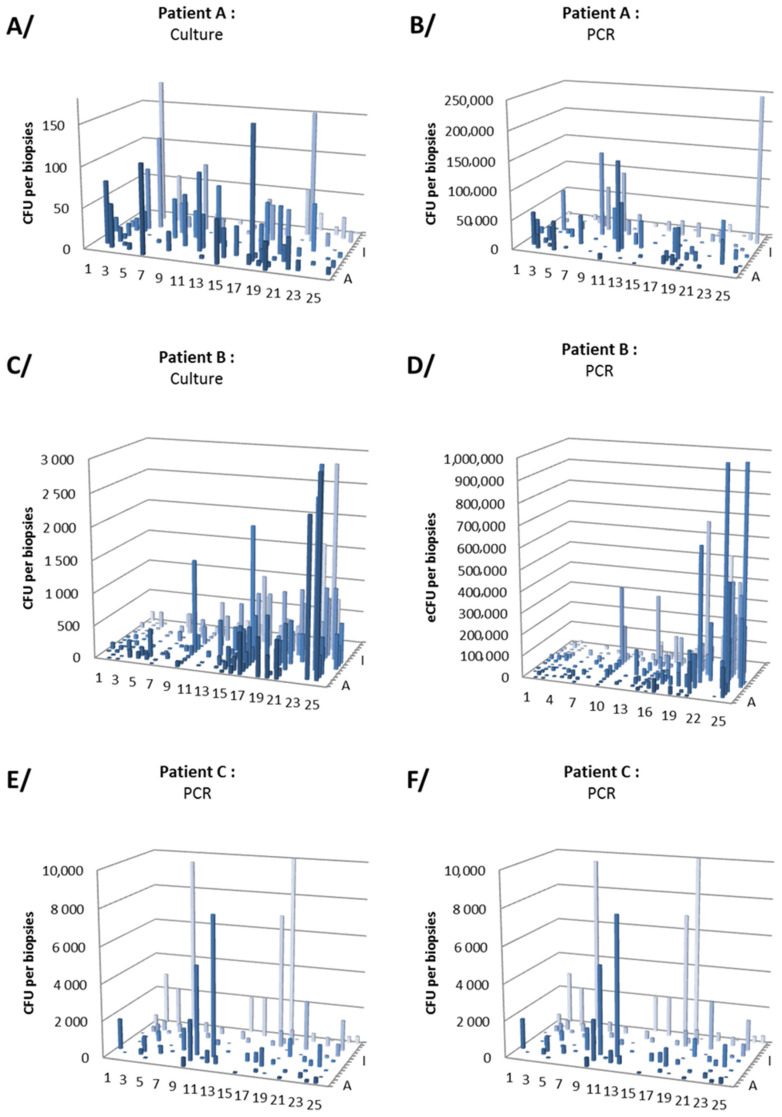
Quantitative distribution of *Helicobacter pylori* in the stomach of three patients. (**A**,**B**) correspond to patient A; (**C**,**D**) to patient B; (**E**,**F**) to patient C. (**A**,**C**,**E**) correspond to bacterial quantification using culture method in CFU/biopsy and (**B**,**D**,**F**) to qPCR method in eCFU/biopsy. Colors are only meant to clarify the visualization of the charts.

**Figure 2:**
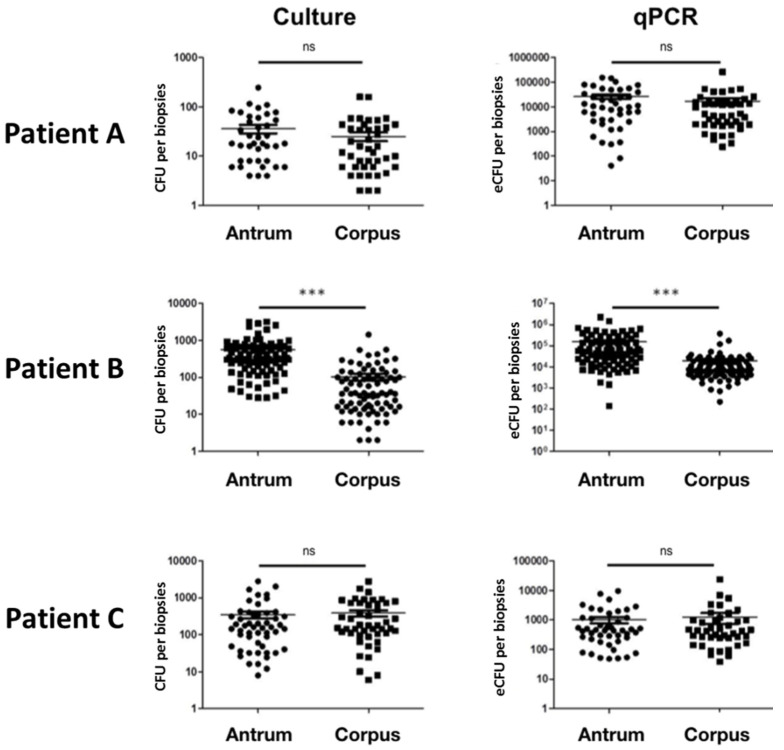
Bacterial load of *H. pylori* in biopsies depending on the quantification method and location. Ns, non-significant. *** *p* < 0.001. Significant difference between antrum and corpus was found by both culture and qPCR for patient B (*p* < 0.001). No difference could be observed for patient A and B (*p* = ns).

To summarize, *H. pylori* was detected in 96.9% (CI_95%_ 94.6–98.3) of biopsies (*n* = 354/365) by culture and 94.9% (CI_95%_ 92.1–96.7) of biopsies (*n* = 351/370) by qPCR of all investigated sites, without any particular location for negative biopsies (Figure 1). Antral density (determined by both qPCR and culture) was significantly higher than in corpus in only one patient (B) out of three (Figure 2).

Considering the surface area of the punches and the surface area of the stomach as estimated by Helander and Fandriks in 2014, the mean number of *H. pylori* bacteria was estimated at an average of 1 × 10^6^ culturable bacteria per stomach [32]. Real-time qPCR counted more than 1.5 × 10^8^ *H. pylori* genomes per stomach (i.e., 150 times more than the culture) with a significant correlation between qPCR and culture counts of all three stomachs (Spearman’s correlation coefficient, *r* = 0.144, *p* < 0.01). Gastric mapping of *H. pylori* infection in the stomach of the three patients showed high variability of bacterial densities, even among close biopsies (Figure 2, Table 2).

### 3.3. Antibiotic Resistance

**Figure 3:**
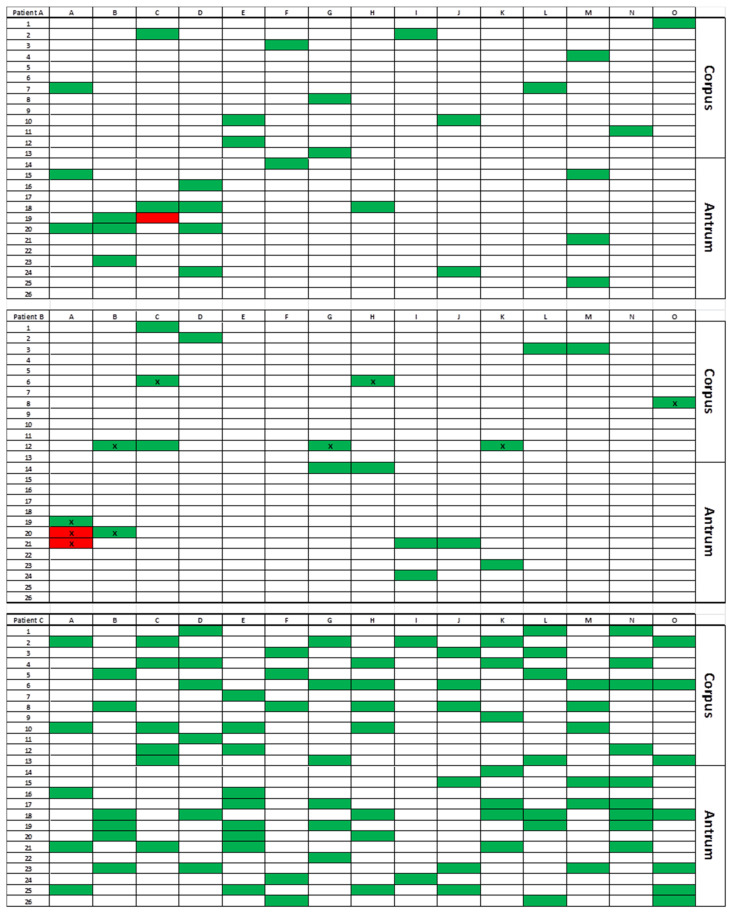
Gastric mapping of resistant and susceptible isolates in collected sleeve gastrectomy. Green squares: Biopsies with isolates susceptible to clarithromycin, metronidazole, amoxicillin, levofloxacin, and tetracycline. Red squares: Biopsies with isolates resistant to clarithromycin for patient A, to metronidazole for patient B. X, *cagA*-positive genotype.

Biopsies were randomly selected and then tested for AST (E-test method) or Scorpion PCR (Figure 3).

For patient A, AST was performed on 44 isolates (25 biopsies) and Scorpion PCR was performed on 118 isolates (including the 44 isolates tested by AST). Only one isolate among the five isolates from a unique biopsy was resistant to clarithromycin by AST (MIC = 8 mg/L) with an A2143G mutation in the 23S rRNA gene detected by Scorpion PCR.

For patient B, AST was performed on 96 isolates (21 biopsies), and Scorpion PCR was performed on 117 isolates (including the 96 isolates tested by AST). Among all these tested isolates, all were susceptible to clarithromycin (by AST and/or Scorpion PCR). Eight isolates (among 15 isolates from two adjacent biopsies) were resistant to metronidazole by AST (MIC > 256 mg/L).

For patient C, AST was performed on 49 isolates (28 biopsies), and Scorpion PCR was performed on 117 isolates (including the 49 isolates tested by AST). All strains were susceptible with a wild-type 23S rRNA gene, considered as discrepant with pre-sleeve biopsy finding the presence of clarithromycin- and metronidazole-resistant strain (i.e., MICs > 256 mg/L and A2143G 23S rRNA gene mutation). Therefore, 84 additional biopsies (38 from corpus and 46 from antrum) were analyzed by Scorpion PCR, and all the post-sleeve testing demonstrated the presence of clarithromycin-susceptible strains. 

### 3.4. RAPD Genotyping and CagA Detection

First, to explore the genetic diversity of isolates present in different locations of the gastric mucosa, RAPD and *cagA* genotyping were performed on one isolate from twenty randomly selected (ten antral and ten corpus biopsies) for each patient. Second, to explore the genetic diversity of isolates present in a single biopsy, from two to twelve isolates (as many as colonies appeared on culture plates) per biopsy for five antral and five corpus biopsies for each patient. A total number of 119, 108 and 119 isolates were genotyped for patient A, B, and C, respectively.

RAPD patterns were clearly distinct between the isolates from the three patients. Both patients A and C were uniformly infected with a unique genotype (characterized by a single RAPD pattern), *cagA*-negative and *cagA*-positive for patient A and C, respectively (Figure 3). For patient A, susceptible and resistant isolates were from the same genotype. For patient C, both the preoperative strain and post-sleeve isolates were also from the same genotype.

For patient B, two distinct RAPD patterns were observed, without a specific location in the stomach. These distinct genotypes presented two different *cagA* genotypes (Figure 3). The *cagA*-negative genotype was dominant with 82 isolates (82/108; 75.9%). The other isolates were *cagA*-positive and presented two different antibiotypes with metronidazole-resistant isolates (*n* = 8/26; 30.8%) and metronidazole-susceptible isolates (*n* = 18/26; 69.2%). No isolate with different genotypes (RAPD pattern and *cagA* genotype) within the same biopsy was observed.

## 4. Discussion

This work is the first, to the best of our knowledge, to exhaustively explore, using culture and molecular methods, the nearly complete surface of the *H. pylori*-infected human gastric mucosa. For ethical reasons, the number of mucosal biopsies obtained during endoscopic procedures is limited, so previous studies (based on culture or PCR) analyzed only five to nine biopsies [16,33,34,35]. Some previous studies based on histological results or limited to detection of urease activity, without bacterial culture or PCR, have analyzed up to eleven or twelve biopsies [13,17]. In our study, gastric sleeve surgical therapy allowed the collection of biopsies for each patient from which 90, 177 and 103 biopsies per patient were tested. More than one-third of the biopsies were analyzed by culture and quantitative real-time PCR and at least 20 biopsies from each patient for genotype determination and virulence characterization (*cagA* status). Only two previously-published studies have explored the whole surface area of *H. pylori*-infected stomachs, in an experimental pig infection or with urease activity detection only [36,37]. In this study, the patchy distribution and the variability of viable bacteria were similar to those described for humans.

Infection by *H. pylori* seems to be extended to the whole gastric surface as a low number of biopsies remained negative (3% by culture and 5% by PCR). This rare absence of detection could be due to the usual default sensitivity of the culture and PCR, rather than to a real absence of the pathogen in these locations. Moreover, no specific area could be highlighted with absence of *H. pylori* detection, suggesting that a single biopsy could be enough for *H. pylori* detection, independently of the considered method, as previously-described results suggested [38,39]. 

Comparison between studies estimating the bacterial load at various sites of the gastric mucosa is very difficult, due to a relatively low number of biopsies per anatomical piece and to very different methodologies. Nevertheless, as in the present study, they demonstrated the highly variable bacterial load per biopsy characterizing the patchy distribution of the organism in the gastric mucosa [4,15,35,36,37,40].

As expected, the bacterial load measured by culture was lower than qPCR quantification (150-times lower) with a positive correlation between these measures [40,41,42]. This difference could reflect a loss of bacterial viability between gastrectomy and freezing of biopsies (limited to one hour for the first biopsies to four hours for the last biopsies) and/or due to thawing before seeding on culture plate. However, no differences between biopsies sampled at the beginning and at the end of the process were observed. This difference could be mostly due to the presence of viable, but non-culturable forms of the bacteria which has been described as coccoid forms of *H. pylori* (Percival FEMS 2009) [43]. The final explanation could consist in a clustering phenomenon, in which several bacteria are inseparable despite vigorous vortex stirring. Several clustered bacteria would then appear as a unique colony, counted as one CFU.

High inter-patient variability of the bacterial load has been observed. The patient with the fewest bacteria was also the one with *cagA*-negative strains only. This result is concordant with other studies that demonstrate a higher bacterial load in case of infection by a *cagA*-positive strain [35,44,45]. Moreover, the intra-patient difference in bacterial density was observed for the patient infected with both *cagA*-positive and *cagA*-negative strains (contrary to the two other patients). Heightened bacterial load in the antrum, observed in one patient, has also been evocated in other studies, with controversial results [13,14,16,17,35,44,45].

RAPD genotyping using two different primers demonstrated multiple infections (different RAPD fingerprinting among the different isolates from a single patient) in one of these patients. Multiple infections have been suggested by many studies, but never in a study analyzing as many biopsies and isolates [19,20,21,22]. A French-Tunisian study demonstrated on one biopsy per patient (42 patients) and three to eleven isolates per biopsy that in a low-prevalence country, such as France, multiple infections remained quite rare (5%), but that they were frequent in a high-prevalence country, such as Tunisia (48%) [19]. A recent study among 16 German patients with up to 30 isolates collected from antrum, corpus and fundus did not find any multiple infections [22]. In the present study, the patient with multiple infections was born in Portugal, a high-prevalence country [46]. 

AST identifies the minor-frequency resistant clones in a large majority of the susceptible population for the three patients. For patient A, only one clarithromycin-resistant isolate was found, located in the upper part of the stomach, close to the lesser curvature (12 mm away from the anterior incision of the stomach performed during the sleeve-gastrectomy). This isolate yielded the same genetic pattern as the other susceptible isolates of the patient, suggesting that the resistant isolate emerged from the susceptible ancestor under antibiotic pressure. For patient B, metronidazole-resistant isolates were detected from two adjacent antral biopsies (6 mm from the anterior incision). In this patient, RAPD genotyping revealed two distinct profiles consistent with *cagA* status. Metronidazole-resistant isolates all belonged to *cagA*-positive clone (representing 24% of the isolates), which also included clones that were susceptible to metronidazole, concluding to a multiple infections. These results lead to suspect the emergence of metronidazole-resistant clones within the population of metronidazole-susceptible and *cagA*-positive clones. For patient C, as presented in the result section, no resistance neither among the 49 isolates from the 28 first biopsies (by AST and Scorpion PCR) nor among the 84 additional biopsies randomly collected on the whole surface area of the operative material was detected by Scorpion PCR. Only the strain isolated from a biopsy taken from the lesser curvature two months before the sleeve-gastrectomy during the preoperative assessment was clarithromycin- and metronidazole-resistant (MIC = 6 and > 256 mg/L, respectively, and A2143G mutation detected by Scorpion PCR), confirmed by RAPD to be issued from the same genotype as all strains isolated from the surgical sample. The discriminatory power of this technique is such that an identical profile indicates a genetic proximity that one can only find in a single individual or in persons of the same family [30]. This observation is difficult to explain, was the mutant present only in the lesser curvature area that stays in place with the sleeve gastrectomy? It is to note that in the other two patients (A and B), resistant isolates were detected close to the lesser curvature site. If this anatomical region is more favorable to the development of cancer, is it also more favorable to the development of resistance? These observations justify the benefit to focus biopsies in this area in order to better detect precancerous lesions and antibiotic resistance [47].

The analyzed patients had never received eradication therapy against infection with *H. pylori* nor antibiotic treatment or proton pumps inhibitors for a year. Even in the absence of prior treatment of eradication, at least one resistant isolate was detected in all patients, in an anatomical location close to the lesser curvature. As confirmed by a negative breath test, the patients were successfully treated after the sleeve-gastrectomy against *H. pylori* infection with treatments guided by the results of the pre-surgery biopsy or with bismuth quadruple therapy (Pylera, Aptalis Pharma, Bridgewater Township, NJ, USA). As a result, the impact of this heteroresistance on the success of eradication treatment could not be correctly evaluated in this study. There are not sufficient data in this study in support of the hypothesis that micro-heteroresistance may, on a large scale in clinical practice, affect *H. pylori* eradication outcomes. However, according to the presented results, there is a reasonable chance of underestimating or missing antibiotic resistance even if a large number of biopsies are taken for antimicrobial susceptibility testing. As this might certainly negatively affect the success of *H. pylori* eradication treatments, these results highlight the benefits of using antibiotic combinations and bismuth in effective eradication treatment and may explain bacterial eradication failure (5–10%) even when the strain isolated is susceptible to antibiotics on one biopsy. While there are no recommendations as to whether one, two or more gastric biopsies need to be taken for the assessment of *H. pylori* resistance to antibiotics, several authors have indicated that multiple gastric biopsies from both the antrum and the corpus should be used to detect antibiotic resistance [47,48]. The presented results could encourage careful sampling of the lesser curvature area.

## 5. Conclusions

Infection with *H. pylori* substantially spread the whole surface of the stomach with a patchy distribution. The three studied stomachs exhibited very low-frequency subpopulations, resistant to at least one antibiotic and hardly detectable in clinical practice. The three reported observations to suggest the interest to biopsy the lesser curvature of the stomach to detect antimicrobial resistance.

## Figures and Tables

**Table 1:** Patient characteristics before sleeve gastrectomy.

Patient	Age (years)	Sex	BMI (kg/m^2^)	Place of Birth	Digestive Symptom	Results on Biopsies Collected Two Months before Sleeve Gastrectomy	
						Histology	AST
A	29	F	43	France	No	Chronic active gastritis	S
B	42	F	49	Portugal	No	Chronic active gastritis	S
C	29	F	48	France	No	Chronic active gastritis	Clari R-Mtz R

F, female; S, susceptible to all tested antibiotics (clarithromycin, amoxicillin, levofloxacin, metronidazole, and tetracycline); Clari R, resistant to clarithromycin; Mtz R, resistant to metronidazole; AST, antimicrobial susceptibility testing.

**Table 2:** Bacterial load estimated by culture and quantitative real-time PCR.

	Patient A	Patient B	Patient C	Total
**Culture** (culture-positive biopsies/analyzed biopsies (mean CFU/biopsy))				
Antrum	38/42 (36)	90/90 (553)	52/52 (349)	180/184 (377)
Corpus	42/46 (25)	85/86 (102)	47/49 (399)	174/181 (171)
Total	80/88 (30)	175/176 (333)	99/101 (373)	354/365 (271)
**qPCR** (PCR-positive biopsies/analyzed biopsies (log mean eCFU/biopsy))				
Antrum	43/44 (4.41)	87/90 (5.18)	47/53 (3.00)	177/187 (4.89)
Corpus	46/46 (4.23)	86/87 (4.28)	42/50 (3.08)	174/183 (4.11)
Total	89/90 (4.32)	173/177 (4.94)	89/103 (3.04)	351/370 (4.67)
